# “One size does not fit all” – lessons learned from a multiple-methods study of a resident wellness curriculum across sites and specialties

**DOI:** 10.1186/s12909-021-02995-z

**Published:** 2021-11-13

**Authors:** Deanna Chaukos, Jonathan P. Zebrowski, Nicole M. Benson, Alper Celik, Emma Chad-Friedman, Aviva Teitelbaum, Carol Bernstein, Rebecca Cook, Afia Genfi, John W. Denninger

**Affiliations:** 1 Mount Sinai Hospital/ University of Toronto Temerty Faculty of Medicine, 600 University Avenue, Toronto, Ontario M5G1X5 Canada; 2 Massachusetts General Hospital/ Harvard Medical School, Boston, MA USA; 3 McLean Hospital/ Harvard Medical School, Belmont, MA USA; 4 Hospital for Sick Children, Toronto, Canada; 5 University of Maryland, College Park, MD USA; 6 NYU Grossman School of Medicine, New York, NY USA; 7 Montefiore Medical Center/Albert Einstein College of Medicine, New York, NY USA; 8 Dell Medical School/University of Texas-Austin, Austin, TX USA; 9 Nava PBC, Washington, DC USA

**Keywords:** Resident wellness, Learning environment, Resident, Wellbeing, Resident wellbeing

## Abstract

**Background:**

There is growing recognition that wellness interventions should occur in context and acknowledge complex contributors to wellbeing, including individual needs, institutional and cultural barriers to wellbeing, as well as systems issues which propagate distress. The authors conducted a multiple-methods study exploring contributors to wellbeing for junior residents in diverse medical environments who participated in a brief resilience and stress-reduction curriculum, the Stress Management and Resiliency Training Program for Residents (SMART-R).

**Methods:**

Using a waitlist-controlled design, the curriculum was implemented for post-graduate year (PGY)-1 or PGY-2 residents in seven residency programs across three sites. Every three months, residents completed surveys, including the Perceived Stress Scale-10, General Self-Efficacy Questionnaire, a mindfulness scale (CAMSR), and a depression screen (PHQ-2). Residents also answered free-text reflection questions about psychological wellbeing and health behaviors.

**Results:**

The SMART-R intervention was not significantly associated with decreased perceived stress. Linear regression modeling showed that depression was positively correlated with reported stress levels, while male sex and self-efficacy were negatively correlated with stress. Qualitative analysis elucidated differences in these groups: Residents with lower self-efficacy, those with a positive depression screen, and/or female residents were more likely to describe experiencing lack of control over work. Residents with higher self-efficacy described more positive health behaviors. Residents with a positive depression screen were more self-critical, and more likely to describe negative personal life events.

**Conclusions:**

This curriculum did not significantly modify junior residents’ stress. Certain subpopulations experienced greater stress than others (female residents, those with lower self-efficacy, and those with a positive depression screen). Qualitative findings from this study highlight universal stressful experiences early in residency, as well as important differences in experience of the learning environment among subgroups. Tailored wellness interventions that aim to support diverse resident sub-groups may be higher yield than a “one size fits all” approach.

**Trial registration:**

NCT02621801, Registration date: December 4, 2015 – Retrospectively registered.

## Background

Residency is an intense and formative time during which the steepness of professional and personal growth curves are augmented by stressful work environments, increased responsibility, rigorous schedules, and varying levels of perceived support or guidance [1]. The negative impacts of resident distress are well described, and residents may experience increased challenges to wellbeing compared to medical students or attendings [2].

There is growing recognition that wellness interventions should occur in concert with an integrated and contextual wellness strategy — one that attends to individual needs, institutional and cultural barriers to wellbeing, as well as systems issues which propagate distress [3]. The COVID-19 pandemic has further highlighted the importance of system supports of physician wellbeing, and that certain sub-groups of physicians may experience disproportionate burden from a universal stressor (i.e. young women) [4].

Here we report the results of a multiple-methods study of a skills-based, peer-facilitated group curriculum that teaches stress awareness, exposes common thought distortions propagated by the culture of medicine, and introduces positive coping skills (mindfulness, adaptive perspective taking). We sought to better understand the subjective experience of residency training across diverse groups of residents in different hospital settings, residency programs, and in diverse specialties, recognizing that the time allocated for formal wellness or stress-reduction curricula is only a small part of residents’ overall experience.

## Methods

### Study participants and intervention

The Stress Management and Resiliency Training Program for Residents (SMART-R) [5, 6] was integrated into the required didactic curriculum for junior (post-graduate year [PGY]-1 or PGY-2) residents in seven residency programs across four specialties (Internal Medicine, Neurology, Pediatrics, and Psychiatry) in three large urban, academic teaching hospitals during the 2015–2016 year. SMART-R is a skills-based stress-reduction program that teaches junior residents to identify their own early stress responses, de-stigmatizes common distressing emotional experiences and environmental stressors unique to junior doctors, and promotes self-efficacy through development of adaptive coping skills and perspectives. This curriculum has been implemented in diverse institutions and specialty residency programs, and it was well received by junior residents [5]. It is adapted and abbreviated from the SMART-3RP (Stress Management and Resiliency Training- Relaxation Response Resiliency Program), which has improved stress-related outcomes in diverse and chronically stressed populations [7–10].

Participants consisted of a sample of residents in these programs who consented to participate in a 12-month study of the curriculum. Residents were independently assigned a clinical rotation order by their residency, which in turn determined whether residents would receive the intervention either immediately upon starting the academic year (intervention arm) or, during the second half of the year (waitlist control arm).

### Data capture

Study participants completed electronic surveys every three months during the 12-month study period via Research Electronic Data Capture (REDCap) [11]. Demographic data were collected via the first survey at the time of study enrollment. The surveys included empirically validated instruments measuring stress and hypothesized stress-modifiers targeted by the intervention: the 10-item Perceived Stress Scale (PSS-10, 10 items, range 0–40, higher scores indicate higher stress), [12] the Patient Health Questionnaire depression screening tool (PHQ-2, 2 items, range 0–6, scores 3 or greater suggestive of depression), [13] the General Self-Efficacy Scale (GSE, 10 items, range 10–40, higher scores represent greater confidence in one’s ability to perform tasks), [14] and the Cognitive and Affective Mindfulness Scale (CAMS-R, 12 items, range 12–48, higher scores signify higher levels of everyday mindfulness) [15]. On each survey, study participants also completed free-text responses to four reflection prompts querying themes addressed in the curriculum such as psychological wellbeing and health behaviors, over the prior three months: 1) Describe your general wellness; 2) Describe your general mood; 3) Describe your recent exercise patterns; 4) Describe your sleep quality. The study was approved by the Partners HealthCare Human Research Committee/IRB, the New York University Langone Health IRB, and the Weill Cornell Medicine IRB. All participants provided written, informed consent. Participants received a $5 gift card for each completed survey.

### Quantitative analysis

All analyses were conducted using R statistical programming language (v 3.6.0), packages brms (v 2.10.0) and rstan (v 2.19.2) for regression, and impute (v 1.58.0) for imputation of missing survey values. Data were extracted from completed surveys during the six months of the study (baseline, three months, and six months) to compare the intervention group to the waitlist. Questionnaires that were missing all responses on a given survey were excluded with other data from that individual. Surveys with one missing question were imputed using k-nearest neighbor methods [16]. For baseline comparisons, we used t-tests to compare stress levels between intervention and control groups [17].

Linear regressions were used to assess the impact of the study intervention as well as the contribution of relevant factors to self-reported stress levels. Ethnicity, race, and marital status were not included in the regression analysis due to significant imbalance in the dataset (Table 1). Similarly, residency specialty and program site information were excluded because not all specialties were represented at all sites, and small group size did not allow for meaningful comparison. We then evaluated the relative importance of several factors (self-efficacy, mindfulness, depression, sex) and the impact of the intervention on self-reported stress levels. Sensitivity analyses conducted including these factors yielded similar results to the model described.

**Table 1:** Demographics of resident study participants

Demographics	Total (*n* = 172)	Sufficient data for analysis (n)
Sex		
Male	59	58
Female	70	67
Chose not to answer	43	17 (not used in regression analysis)
Residency Specialty		
Psychiatry	43	38
Internal Medicine	91	68
Neurology	19	17
Pediatrics	19	19
Age Range	25–49	25–49
Ethnicity/Race		
Hispanic	5	4
Asian	27	25
Black/African American	4	4
White	93	89
Other	3	3
Chose not to answer	43	17 (not used in regression analysis)
Marital Status		
Never Married	86	83
Married	40	39
Divorced	3	3
Widowed	0	0
Chose not to answer	43	17

### Qualitative analysis

Utilizing content analysis methodology, [18] three study investigators performed iterative coding of participants’ free-text entries in response to all wellbeing questions. Each coder independently reviewed all entries and generated a list of open codes, while grouping these codes into categories and sub-categories to capture identified themes using an inductive and deductive approach. The three independent codebooks were then reconciled via consensus discussion among coders and into a single codebook. The coders used the consensus codebook to independently re-code 25% of the general wellness question entries, which yielded overall agreement of 68% (compared to goal of 70%). A consensus discussion on entries with code disagreement resulted in further refinement of the codebook. The investigators then jointly coded the next 25% of entries, addressing discrepancies in real time with further codebook refinements. This resulted in > 90% agreement during further rounds of joint coding, with any remaining discrepancies resolved via consensus discussion. This final codebook was then applied by the coders to all entries.

Codes were aggregated and analyzed to assess for themes across the entire study population, and by subgroup (those factors determined to be significant based on quantitative modeling). For the purpose of analysis, non-categorical variables were separated based on literature recommended cutoffs: for the GSE, high self-efficacy and low self-efficacy were distinguished by the median score [14]; for the PHQ-2, positive depression screens were specified as a score of three or greater [12]. For each divided subgroup, code prevalence ratios were compared, and the codes representative of the largest difference within the groups were identified. Using the factors impacting stress identified in the regression model, differences in qualitative themes reported by residents separated by these factors were explored. The codes representing the largest proportional differences comparatively across subgroups were identified and explored.

## Results

### Study population

Of a potential 182 participants, 172 (94.5%) residents consented to participate in the study. Six residents dropped out of the study immediately after enrollment, 77 residents were in the intervention group, and 89 were in the waitlist control group. Of those in the intervention group, six completed zero surveys, 21 completed one survey, 16 completed two surveys, and 34 completed all three surveys during the first six months of the study. Corresponding responses for the waitlist group were 18 completed zero surveys, 19 completed one survey, 19 completed two surveys, and 33 completed all three surveys. One hundred and fifty-one (87.9%) participants (74 in the intervention group and 77 in the waitlist group) completed at least one free-text written reflection. Diary entries ranged from one to 172 words and consisted of one-word phrases to full paragraphs.

### Regression model for stress

A two-sample t-test of stress levels (PSS-10 scores) at time of study enrollment showed no difference (*p* = 0.435, Cohen’s d = 0.139) between the intervention and waitlist control group.

Time course simple linear regression modeling of survey and demographic data indicated that assignment to the intervention group was negatively correlated with stress levels during the 6-month intervention period. However, the estimated coefficients showed a wide distribution of values (Table 2). Therefore, we could not conclude definitively that our intervention was effective in reducing stress. Depression was positively correlated with self-reported stress levels while male sex and self-efficacy were negatively correlated.

**Table 2:** Estimated coefficients and upper and lower confidence intervals of linear regression analysis (Row descriptors from top to bottom: Intercept; Waitlist control; Event refers to the survey timepoint; Self-Efficacy refers to the GSE score; Depression screen refers to the PHQ2 score; Mindfulness refers to the CAMSR score; WLC:event refers to the survey timepoint and WLC interaction term)

	Estimate	Est.Error	CI 95%	CI 95%
**Intercept**	23.84	1.69	20.52	27.15
**Waitlist control (WLC)**	−0.39	0.87	−2.09	1.31
**Event**	−0.32	0.31	−0.93	0.29
**Self-Efficacy**	−0.16	0.06	−0.27	−0.04
**Depression screen**	1.17	0.16	0.85	1.48
**Mindfulness**	0.09	0.05	−0.01	0.18
**Sex Male**	−0.78	0.36	−1.47	−0.07
**WLC:event**	0.48	0.44	−0.37	1.34

### Qualitative analysis

Qualitative content analysis of 1595 self-reflection entries illustrated that, when asked about their wellbeing, junior residents most commonly described sleep deprivation, long work hours, and rotation-related stressors. Residents commonly described an interplay between busy rotations, lack of sleep, and resultant neglect of other areas of their lives:

*… the last two months have been two of the most difficult work months of my life while on inpatient medicine. I was working 80-90 hours per week and sleeping 5-6 hours per night, which made it difficult to find time to do the things I enjoy.*

Residents who had a positive depression screen were more likely to describe a perceived underperformance at work, including self-criticism and feeling overwhelmed, over-stretched, scattered, or worried about making or having made mistakes at work. These residents also described experiencing stressful life events outside of work such as emotional, financial, or personal stressors. One resident described struggling with,

*… lack of sleep and lack of positive reinforcement, not doing the job I wish I was doing. Not feeling competent.*

On the other hand, residents who screened negative for depression were more likely to describe positive and meaningful work experiences, including feelings of accomplishment and engagement, personal growth, connection with colleagues, and finding their role on a team. One resident described,

*[I have been] generally well. I've enjoyed the past two months on internal medicine, despite long and difficult hours. I think working alongside great people with good team dynamics made a big difference.*

Another resident noted,

*I feel lucky to be a doctor in [this city]… I feel very fulfilled by my job, and that I’m constantly improving.*

Residents who scored high in self-efficacy were more likely to describe engagement in physically healthy behaviors (exercise, healthy eating, and tracking health goals), as well as maintaining positive connections to others. One resident described,

*I am feeling physically well though [there’s] not always enough time for exercise. Professionally feeling quite well, at times challenged by the workload or schedule but enjoying the work. Socially at times a bit lonely, but with a core good group of friends and family, [there is] always someone there when I need them.*

Residents with lower self-efficacy were more likely to describe dealing with a chronic medical illness or injury, a neutral mood, and/or feeling a lack of control at work. One resident described feeling,

“*overwhelmed, feeling like all the important things [in life] are out of my control”.*

Male residents were more likely to describe adaptive perspectives (acknowledging the negatives, while emphasizing the positives) and other positive coping strategies (optimism, gratitude, noting how they were able to exercise control in otherwise uncontrollable situations). Male residents were also more likely to describe physical experiences of stress (including fatigue, or the perceived need to feel awake or energized). One male resident described,

*[I’m] getting reasonable amount of sleep, decent amount of exercise. Emotionally doing well. Feel in control at work and feel like my work is worthwhile.*

Another male resident described,

*I have felt emotionally and physically exhausted from my work, [and from] not [having] much time outdoors or outside of work… I am looking forward to some relaxation time coming up.”*

Female residents were more likely to describe relationships with others (both maintaining connections, as well as difficulties due to being separated from loved ones). Female residents were also more likely to describe feeling a lack of control at work:

*[I*’*m] preoccupied but happy-- worried about my family and friends coping with [an] unexpected loss, but also worried about the future…*

Another female resident noted,

*… My mood is better because I have enjoyed my rotations more, felt connected to my peers and faculty, and spent more time with family.*

## Discussion

Stressors endemic to the culture of medicine have been a focus of prior studies, as have initiatives to tackle physician distress and to promote wellbeing. Here we present results from a multi-site and multi-specialty waitlist-controlled study of a brief wellness curriculum aimed at reducing resident stress. Our study assessed the effect of a brief wellness curriculum in diverse training environments while simultaneously exploring the subjective experience of junior residents. While participating in the intervention appeared to decrease stress over time compared to being waitlisted, we could not definitively quantitate this difference given high data variation.

Nevertheless, our results highlight the heterogeneity of experience among residents, specifically that certain groups (male sex, those high in self-efficacy, and/or without depression) are less likely to report stress. Furthermore, the subjective experience of well-described stressors in the learning environment can be distinctly different between subgroups. Though most residents described common and expected stressors – sleep deprivation, work hours, and rotation-related concerns – residents who reported higher stress not only described these stressors differently but also faced challenges to their wellbeing that were not described as frequently by others. Residents with lower self-efficacy, those with a positive depression screen, and/or female residents were more likely to describe experiencing a lack of control over work.

Though aspects of the learning environment such as rigorous and inflexible schedules or loss of autonomy are commonly described as detrimental to physician wellbeing, [19] our findings indicate that these environmental factors may impact certain residents more than others.

Notably, female residents were more likely to describe both positive and negative impacts on wellbeing related to relationships, whether maintaining connections or managing interpersonal conflict. Male residents, on the other hand, were more likely to describe physical manifestations of stress and to focus on their individual coping. These observations suggest that gendered societal expectations of men and women influence manifestations of stress in medical training. Prior research indicates that relationships during residency are significantly affected by shifting professional identity, and stressors in this sphere are a source of burnout and stress [20]. Residents attending to familial or other external interpersonal obligations are perhaps more likely to experience dissonance between professional goals and their relationships. This finding is consistent with literature demonstrating that women in medicine are at higher risk of burnout or depression, [21] including during residency, [22] and face unique added obstacles impinging on their success in academic medicine [23–26]. The COVID-19 pandemic has heightened aspects of gender inequity in medicine, perhaps especially affecting women physicians with care responsibilities to loved ones [27].

Residents with a positive depression screen were more self-critical and more likely to describe negative personal experiences outside of work. In contrast, residents with a negative depression screen were more likely to describe meaningful work experiences, regular exercise, as well as describe mental states related to wellbeing. While it is not surprising that residents experiencing depression will have more self-deprecating thoughts, and even anhedonia, this study strengthens prior findings that depression may interfere with a physician’s ability to experience worth from their professional activities [28, 29]. Furthermore, physicians experiencing personal life stressors outside of work are more likely to suffer from depression or anxiety [30].

Residents with higher self-efficacy more frequently described engaging in positive health behaviors (e.g., exercise, healthy eating, maintaining connections, and tracking goals). In contrast, residents with lower self-efficacy were more likely to describe coping with a chronic illness. Self-efficacy is a character trait that is highly valued in medicine and is often treated as an intrinsic personal attribute. However, our data suggests that self-efficacy is also influenced by circumstance: an individual coping with a chronic illness, for example, may differently perceive their ability to change aspects of their experience.

This study has several limitations. First, the study intervention was embedded within didactic curricula in a diverse group of residency programs in different environments. As a result, we could not account for many potential confounders arising from heterogeneity of sites and site-specific implementations of the curriculum. Second, the study surveys measured variables hypothesized to decrease stress and improve wellbeing, [31] but our study did not include an independent measure for wellbeing or resilience. Third, not every specialty was represented at each site, which prevented us from making meaningful comparisons across specialties/sites. Similarly, we could not evaluate the impact of race or marital status because of the low numbers of racial minority or married residents represented in the study population. Finally, although 85% of participants completed at least one survey, there was a significant amount of missing data, which may lead to selection or response biases.

## Conclusions

This multi-specialty and multi-site study of junior residents demonstrated that certain factors (male sex, self-efficacy, and depression) influence junior residents’ perceived stress and that stressors in the learning environment may be experienced differently by subgroups of residents. The qualitative reflections elucidate the differences in subjective experience of residents in these diverse subgroups, including those at higher risk for distress. Though the intervention was not significantly linked to decreased stress, practical and embedded initiatives like the SMART-R, when included as part of a robust multi-pronged wellness strategy, may help us make implicit inequities in the learning environment more explicit, and over time, shift culture. Further, there is perhaps opportunity for medical educators and residency wellness support systems to consider specific primary prevention and secondary screening strategies for residents more likely to experience distress (i.e. providing informational resources/supports for residents in caregiving roles at orientation) [32]. Perhaps a comparable analogy is the concept of a ‘culture of safety’ in the field of healthcare quality and safety where there is the recognition that there is no single mechanism or set of fixed interventions that can improve patient safety. Instead, it is essential to create a local culture which fosters open exchange of safety concerns and flexibility to respond to issues as they arise. What is clear from this study is that a “one size fits all approach” to wellness initiatives is unlikely to be effective for all trainees [32]. It is essential that we attempt to understand the diversity of the physician workforce as we work to make changes to the medical learning environment.

## Data Availability

The datasets generated and analysed during the current study are not publicly available due to concerns about potential compromise of individual privacy, but are available from the corresponding author on reasonable request.
